# Histological Changes on Liver Glycogen Storage in Mice (*Mus musculus*) Caused by Unbalanced Diets

**DOI:** 10.4137/cpath.s505

**Published:** 2008-04-23

**Authors:** Esma Ulusoy, Banu Eren

**Affiliations:** Faculty of Arts and Science, Department of Biology, University of Ondokuz Mayıs, Samsun, Turkey

**Keywords:** liver, high protein diet, glycogen, unbalanced diet

## Abstract

Weight-losing diets have appealed to people who want to lose weight in the short-term. They usually apply high-protein (HP) diets (like Atkin’s, Stillman’s, Scarsdale) which they practice for 2 weeks or so. Unfortunately, these people who have rapid weight loss return to their old habits and quickly regain the weight lost. We have shown in previous work that actually these weight losses have been associated with body fluids, protein and glycogen storage. In our study, we examined the effect of unbalanced diet—related to an HP diet- on liver glycogen storage.

For this study 40 Swiss albino mice consisting of two groups were used. The first group (HPSD) was fed with 25% HP for fifteen days and then were fed standard meals for the remaining 15 days; the other group was fed with standard meals throughout. The two groups were fed their respective diets for 30 days. At the end of 15th, 20th, 25th and 30th days 5 from each group were killed with cervical dislocation. The livers were removed perfused and then fixated.

There were major differences in weight between the first and the fifteenth days. We detected remarkable increase in the weight gain of mice in the remaining 15 days. Glycogen storage was significantly reduced in HPSD (15) stained with PAS. In the others 20th, 25th and 30th days abnormally dense glycogen deposits were observed. Vacuoles in the hepatocyte cytoplasm, brownish deposits within hepatocytes, wide sinusoids, macrovesiculler steatosis structures and hydropic degeneration were observed in PAS and H&E stained HPSD group.

As a result for the HPSD group a significant decrement in glycogen storage at the 15th day and also an accumulation of excessive amounts of glycogen deposits in mice liver was observed in the normal feeding phase.

**A single liver cell** can carry out more than 500 separate, specialized metabolic activities (Solomon et al. 1999). Among these specialized activities the most significant one is glycogen storage. The decrease or increase of glycogen storage of the liver is related to the diet of the subject. (Andreoli et al. 2000). Increase or decrease of the glycogen storage in the liver depends on nutrition. This control is maintained by the operation of the insuline-glucagon hormones in the body.

## High-protein diets and weight reduction

High-protein diets are not recommended because they restrict healthy foods that provide essential nutrients and do not provide the variety of foods needed to adequately meet nutritional needs. Individuals who follow these diets are therefore at risk of compromised vitamin and mineral intake, as well as potential cardiac, renal, bone, and liver abnormalities overall (Jeor et al. 2001). However, there exist very few studies about the effects of unbalanced nutrition on liver glycogen storage. But, the significant decrement in glycogen storage on mice that was fed for 30 days with 25% high protein diet have been found us (Ulusoy and Eren, 2006).

After 30 days without food, plasma glucose and free fatty acids of *Phrynops hilarii* did not change significantly, whereas plasma insulin, estimated by a mammalian immunoassay method, decreased to about 40% of fed levels and increased 5-fold after re-feeding for 72 hr (Silva and Migliortni, 1990). So that unbalanced diet effects coordinating between insulin and glucagon secretion.

## Role of insulin in the diets

Consumption of carbohydrates triggers release of insulin from beta cells. Alpha cells become inhibited and cease to secrete glucagon. Taken together, these actions produce a rapid return to fasting blood sugar levels and storage of glucose as glycogen or lipid. A protein-rich meal leads to release of both insulin and glucagon. The latter stimulates gluconeogenesis and release of the newly formed glucose from the liver to the blood stream. The very moderate rise in insulin associated with the protein meal stimulates uptake of the sugar formed in the liver by muscle and fat tissue (Franz, 2000).

High-protein diets typically offer wide latitude in protein food choices, are restrictive in other food choices (mainly carbohydrates), and provide structured eating plans. They also often promote misconceptions about carbohydrates, insulin resistance, ketosis, and fat burning as mechanisms of action for weight loss (Jeor et al. 2001).

Unfortunately, 70% of successful weight losers return to their old habits and within 2 years regain at least half of the weight lost. These patients typically have little insight into the reasons why the weight was regained, and consider themselves ‘failures’ to traditional diet programs. They become prime targets for diets promising rapid and easy weight loss (Denke, 2001).

Several diets promise that, as long as you restrict carbohydrates, you will lose weight and you can eat as much food as you want. There may be a kernel of truth to this claim. For some patients, high-protein intake suppresses appetite (Johnstone, 1996). For other patients, ketosis from carbohydrate restriction suppresses appetite. Restricting carbohydrate eliminates some popular foods that are often consumed in excess such as bread, cereal, soft drinks, french fries, and pizza. By simply excluding carbohydrate foods, patients following the Atkins diet typically consume 500 fewer calories a day (Yudkin, 1960).

Apart from this research we observed negative effects on liver glycogen storage on the metabolism in those who are on high protein diets (Ulusoy and Eren, 2006). For this reason the aim of this research is to identity histologicaly the change dependence at term in the liver glycogen storage of the mice switching habitual diets (15 days) after having HPdiet (15 days).

## Methods

Forty male Swiss albino laboratory mice (*Mus musculus*), 60–80 day-old, average initial body weights 30–40 g, were used in this study. The animals were housed in individual wireless steel cages under standardized conditions of temperature and light.

The animals were divided into two groups. The first group was the one which was fed with HP diets (casein) for 15 days and then was fed their standard diet for the remaining 15 days; the other was the control (C) group that was fed with the standard diet. All the mice were given 12 ± 2 g diet per day in separated cages. While the control group was given a standard diet the other was fed with 25% HP diet (Reeves et al. 1993). HP diet used on mice was prepared by powder (Table 1). This powder was made up of fat-free casein and was gluten-free. As 2.5 g powder was equal to 2.2 g protein; this 25% HP was prepared, diluting distillated water 6.3 ±1 +2.5 g powder.

The animals had free access to water and were fed the diet for 30 days. At the end of 15th, 20th, 25th and 30th days, 5 from each group were killed by cervical dislocation after being weighed. The livers were perfused with saline solution (0,9%) injected through main hepatic artery and then removed. Later the liver tissues were fixated with 80% ethanol for 12 hours. The fixated liver tissues were embedded in paraffin and cut into 5 μm sections and then stained with H&E and PAS (Bancroft and Stevens, 1996).

In our study, we used analytically pure substances supplied by Sigma-London Company, standard diet was supplied by Samsun Yem Company and the protein which was used to prepare the HP diet was supplied by Nutricia Cuijk-Holland Company.

Data gathered was examined in five different periods for normal distribution. For data a normal distribution analysis of variance (ANOVA) was used. Statistics were performed by the statistical package SPSS for Windows. The limit of significance was set at p < 0,05.

## Findings

**Figure 1.**
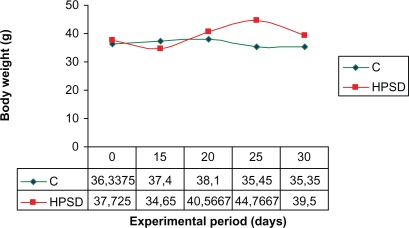
Weight curves of mice, fed high protein (HPSD) and normal(C) diets. Body weights of 30 day old mice.

In the HPSD group weight loss was observed in the mice of 15th days when compared to the 1st day and also to the control group. The weight loss between the 1st day and the 15th day was meaningful (P < 0,05). However, there was statistically significant weight difference between 15th day and the 30th day (P < 0,05). The significant increase in weight afterwards was observed (20th, 25th and 30th) (P < 0,05). There was not a significant weight decrease or increase in the control group (Fig. 1).

**Figure 2.**
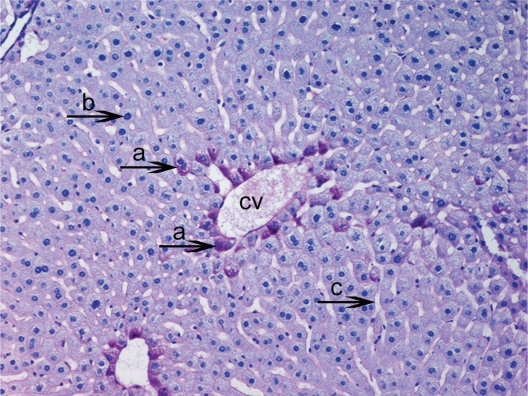
Light micrograph of the histological view of livers in C (15). Observe glycogen deposits in the hepatocyte cytoplasm (**a**) around the central vein (cv). The nucleus of hepatocyte (**b**) and sinusoids (**c**). These accumulations are seen as pink areas of PAS-positive material throughout the section. PAS × 200.

**Figure 3.**
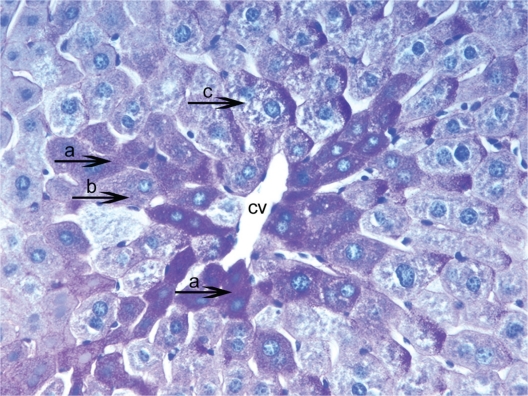
Light micrograph of the histological view of livers in hepatocytes glycogen deposits and vacuolation was seen in excessive amounts from HPSD (25). Observe densely glycogen deposits in the hepatocyte cytoplasm (**a**) around the central vein (cv). Showing a remarkable increase of hepatocytes swelling (**b**) and small vacuoles (**c**). These accumulations are seen as pink areas of PAS-positive material throughout the section. PAS × 400.

**Figure 4.**
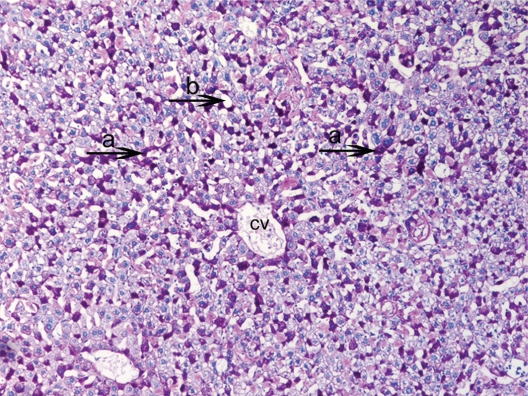
Light micrograph of the histological view of livers in HPSD (30). Observe the accumulation of excessive amounts of glycogen deposits (**a**) in all liver. They correspond to the pink-purple stained areas. Also central vein (cv) and width sinusoids (**b**) PAS × 100.

**Figure 5.**
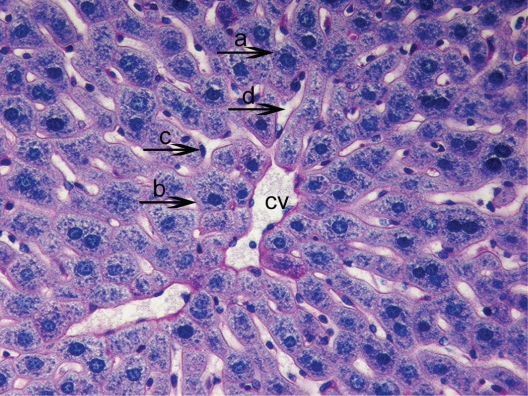
Light micrograph of the histological view of livers in HPSD (25). The hepotocytes that is not accumulated glycogen into them have very dense granulated (**a**) Observe the swelling hepatocytes (**b**) and kuppfer cells around the central vein (cv). PAS × 600.

Firstly, that glycogen deposits on the fifteenth day in control group were obsereved (Fig. 2). PAS technique allowed us to observe a significant decrease in glycogen storage on 15th day of HPSD group. Glycogen was found in minimum amounts around central vein and periportal areas. The following days in hepatocytes glycogen deposits and vacuolar hydropic degeneration was found in excessive amounts from 20th (Fig. 3) to 30th (Fig. 4). The hepatocytes that were not accumulating glycogen were very densely granulated (Fig. 5).

**Figure 6.**
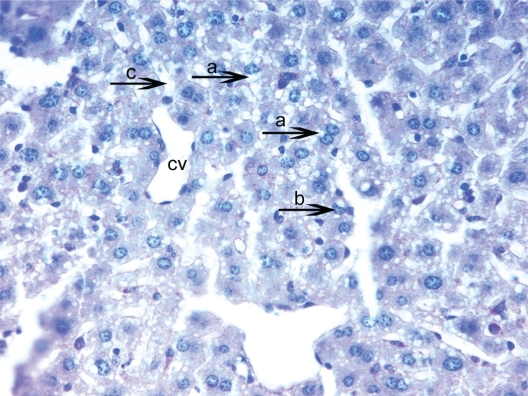
Light micrograph of the histological view of livers in HPSD (20). In PAS staining macrovesiccular stetosis structures (**a**) in liver tissues is detected and these are pushed the nuclei peripheral areas (**b**) Showing a remarkable degenerate sinusoids. PAS × 600.

**Figure 7.**
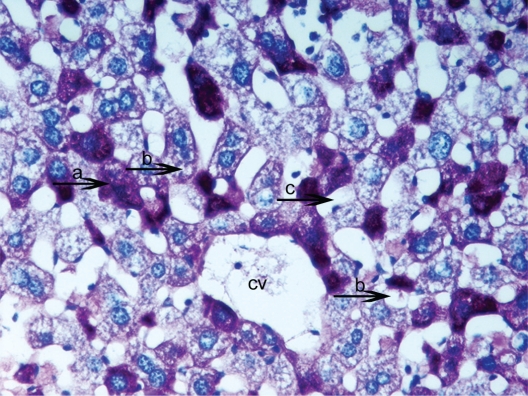
Light micrograph of the histological view of livers in HPSD group (25). Very dense glycogen deposit within hepatocytes (**a**), a large amount of dilatation in sinusoids (**c**) and a small amount of vacuolar hydropic degeneration (**b**) appear. PAS × 500.

**Figure 8.**
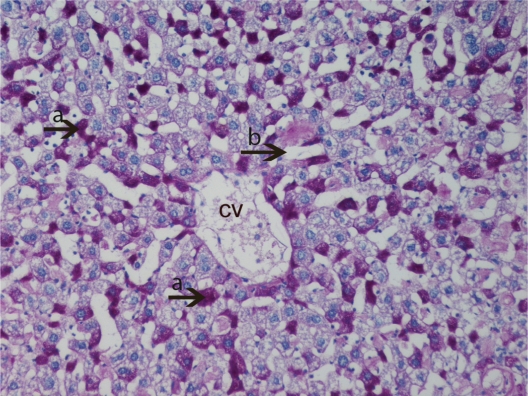
Light micrograph of the histological view of livers in HPSD group (15). Observe the exessive amount of glycogen deposits (**a**) around the central vein (cv). Much more vacuolar hydropic degeneration (**b**) and significantly width sinusoids (**c**) are observed. PAS × 200.

In PAS staining, macrovesicular steatosis structures in liver tissues were detected and these were pushed into the nuclei peripheral areas (Fig. 6). A large amount of dilatation in sinusoids and a small amount of vacuolization was detected in HPSD (25) (Fig. 7). But on the 30th day in HPSD much more vacuolar hydropic degeneration was observed (Fig. 8).

### Protein intake and hormon regulation

We can explain the HPSD diet group’s loss of weight in the first weeks (8.2%) and the later weight gain exceeding the initial body weight (4,7%) as follows:

Patterns of plasma hormones induced changes in insulin resistance and nutrient partitioning between dam and fetus. Whereas the low protein diet did not support nutritional requirements, the medium protein diet fulfilled the competing maternal and fetal demands for growth (Jimenez-Gancedo et al. 2004). Resurgence of low carbohydrate diets has been fueled by rising obesity and insulin resistance in the general population. Although the Atkins’ Diet is the prototype of the low carbohydrate diet, The Sugar Busters Diet, Carbohydrate Addicts Diet, Protein Power Diet, and the Zone Diet are all variations on this common theme (Denke, 2001). In our studies a significant weight gain and glycogen accumulation HPSD diet groups was observed, compared to control groups.

Modest increases of systemic insulin concentrations can suppress free fatty acid mobilization from adipose tissue and thereby increase hepatic sensitivity to portal insulin. Also, concentrations of insulin are higher in portal than in systemic plasma, and the liver may be more sensitive than muscle to the effects of insulin (Rebrin et al. 1995; Lewis et al. 1996). Modulation of insulin secretion during fasting consequently regulates hepatic glucose production and thus the concentration of glucose in plasma. Small changes in plasma glucagon also modulate hepatic glucose production, in effect by altering hepatic responses at given levels of insulin (Myers et al. 1991; Jiang and Zhang, 2003). Insulin resistance can lead to a reduction in glycogen synthesis (Radziuk, 1982) whereas elevation in the catecholamine levels can contribute to increased glycogen degradation (Kuwajima et al. 1986; Fedatto-Junior et al. 2002). Thus, high protein diet promotes the onset of diabetes (non-obese-diabetics) (Schneider et al. 1996). All the above observe that in this type of unbalanced diet there has been an improvement of insulin resistance.

### Unbalanced diet triggers hormon balanced

In our study, the unbalanced diet especially caused weight gain and abnormal glycogen deposits. Also Steppan et al. (2001) reported that obesity is a major risk factor for insulin resistance and type 2 diabetes mellitus. Adipocytes secrete numerous substances that might contribute to peripheral insulin sensitivity. The discovery of an adipose-specific secreted protein called ‘resistin’ which circulates in the mouse, with increased levels in obesity, and has effects on glucose homeostasis that oppose those of insulin.

Because insulin, adrenaline, noradrenalin and cortizon can be accepted as the hormones that affect the fat cell, it was reported in various studies that while insulin fasten lipogenesis in cells, others activate lipolysis (Caro et al. 1996; Steppan and Lazar, 2002). In another study, it was reported that, insulin resistance had developed in all mice models which had become obese following a fructose diet (Shuldiner et al. 2001). These results support the significant increase of weight in HPSD group in our study.

In our study, the unbalanced diet especially caused weight gain. Also Steppan et al. (2001) reported that obesity is a major risk factor for insulin resistance and type 2 diabetes mellitus. Adipocytes secrete numerous substances that might contribute to peripheral insulin sensitivity. The discovery of an adipose-specific secreted protein called resistin which circulates in the mouse, with increased levels in obesity, and has effects on glucose homeostasis that oppose those of insulin.

Indeed, counter to the current U.S. Dietary Guidelines which promotes diets high in complex carbohydrates, recent clinical investigations support the efficacy of high-protein diets for weight loss/fat loss, as well as for improved insulin sensitivity and bloodlipid profiles. Thus, the popularity of high protein diets for weigh loss is unquestionable. However, there are always some concerns about high-protein diets (Manninen, 2004).

Popular books and news stories have encouraged individuals to avoid carbohydrate-rich foods, suggesting that high-protein foods will not stimulate insulin release. Contrary to this popular myth, however, proteins stimulate insulin release, just as carbohydrates do. Clinical studies indicate that beef and cheese cause a larger insulin release than pasta, and fish produces a larger insulin release than popcorn (Holt et al. 1997). This situation caused us to consider that when mice returned to their habitual diet after the HP diet, insulin resistance developed in cell because of abnormal insulin secretion activity

In this context we had mentioned that the insulin resistance had occurred in the animals fed with a high protein diet, whereas elevation in the catecholamine levels can contribute to increased glycogen degradation (Kuwajima et al. 1986; Fedatto-Junior et al. 2002).

### The relation of insulin resitance and resistin

Adipocytes secrete several substances such as leptin, Acrp30, TNFα, adipsin, IL-6, plasminojen activator-inhibitor, transforming growth factorβ, angiotensinogen, metallothionein and resistin (Jequier and Tappy, 1999). Resistin might contribute to peripheral insulin sensitivity and insulin resistance. Type 2 diabetes characterized by target-tissue resistance to insulin, is epidemic in industrialized societies and is strongly associated with obesity. Resistin is a potential link between obesity and insulin resistance (Uysal et al. 1997; Berger, 2001; Steppan et al. 2001).

In the HPSD groups that we have observed, as well as the storage of concentrated glycogen, dense vacuolar images were seen. According to Kumar (2000) in the situations the ion imbalance and insufficient homeostasis of cells swelling is one of the forms of cell damage. He declared that this situation is the first symptom of cellular destruction and although it’s hard to notice with a light microscope it is more observable in an organ. He said that if all cells are affected, the color of the organ fades and the weight of it increases with turgor. He reported that in a microscopic view it is possible to see small and clear vacuoles in cytoplasm and this kind of non-fatal reversible change causes hydropic changes. Reports support swelling, also vacuolization in cells and hydropic degenerations found in this research.

In our research, steatosis of liver was observed in HPSD groups. This is also supported with other studies. It was reported that the deficiency of protein-calorie, fasting, rapid weight loss and gastrointestinal surgeries cause steatosis. The causes of steatosis are reported as protein-calorie deficiency, hunger, rapid weight loss, gastrointestinal surgeries (for obesity) (Angulo, 2002). Especially, Research done by Palmer and Schaffner, showed that in overweight adults without primary liver disease, a weight reduction of greater than or equal to 10% results in abnormal hepatic tests (Palmer and Schaffner, 1990) and hepatic steatosis formation (Nomura et al. 1987). The primary metabolic abnormalities leading to lipid accumulation are not well understood, but they could consist of alterations in the pathways of uptake, synthesis, degradation, or secretion in hepatic lipid metabolism resulting from insulin resistance (Angulo, 2002).

We observed before that high-protein diet and low-carbohydrate nutrition cause energy loss (Ulusoy and Eren, 2006). In addition to this we had also observed very dense glycogen storage, vacuolization and cell-swelling in HPSD group hepatocytes. Research done by Haussinger (1996) supports our findings; Insulin also stimulates Na^+^}K^+^}2Cl^−^ co-transport, Na^+^}H^+^ exchange and the Na^+^}K^+^-ATPase in hepatocytes [110 ± 112]. The resulting intracellular accumulation of Na^+^, K^+^ and Cl^−^ causes cell swelling, which induces an activation of glycogen synthase via a PI3-kinase-dependent mechanism. Cell swelling is further promoted by the insulin-stimulated Na^+^-dependent uptake of amino acids (Graf and Haussinger, 1996). These results support the dense glycogen deposits in HPSD group in our study.

## Summary

The amount of resistin dramatically increased in obese mice that are on a diet within 8 weeks. The amount of resistin is high in mice that have obesity and insulin resistance. Resistin might regulate body fat index with a negative feedback and periphery effect. (Steppan and Lazar, 2002). Resistin is thought to be a hormone which has serves like insulin antagonist which has an effect on glucose metabolism. Due to the fact that its receptor isn’t known yet, target cells and tissues haven’t been assigned. However, liver and muscles might be the target organs (Ling et al. 2001). There is a high co-relation between obesity and insulin resistance. If there is high insulin resistance in an obese, here we can definitely mention a disease with many factors and which is related with many genes (Steppan and Lazar, 2002; Shuldiner et al. 2001).

**Conclusively**, our research showed that the mice fed with high protein diet for 15 days faced more serious problems when they are back to their normal diet. Abnormal glycogen storage and serious degeneration observed in the livers of these mice which lose and gain weight very fast, shows how important informed medical advice is when selecting a diet for weight loss.

## Figures and Tables

**Table 1.** High Protein diet used on mice was prepared by powder that is concentrated protein source based on milk protein and soy lecithin(Protifar).

**Average analysis per 100 g**			
Energy	1570kJ/370kcal	Mineraller	
Protein (95,6 En%)	88,5 g	Na	≤40 mg
Carbohydrate (0,5 En%)		K	≤90 mg
Of which sugars		Ca	1350 mg
(lactose)	≤1 g	P	700 mg
Fat (3,9 En%)	≤2 g	Mg	≤30 mg
Fibre	−g	Cl	≤120 mg

## References

[b1-cpath-1-2008-069] Andreoli TE, Carpenter CCJ, Griggs R, Loscalzo J (2000). Cecil Essentials of Medicine.

[b2-cpath-1-2008-069] Angulo P (2002). Nonalcoholic Fatty Liver Disease. N. Engl. J. Med..

[b3-cpath-1-2008-069] Bancroft JD, Stevens A (1996). Theory and Practice of Histological Techniques.

[b4-cpath-1-2008-069] Berger A (2001). Resistin: a new hormone that links obesity with type 2 diabetes. BMJ.

[b5-cpath-1-2008-069] Caro JF, Sinha MK, Kolaczynski JW, Zhang PL, Considine RV (1996). Leptin: The tale of an obesity gene. Diabetes.

[b6-cpath-1-2008-069] Denke MA (2001). Metabolic effects of high-protein, low-carbohydrate diets. Am J Cardio.

[b7-cpath-1-2008-069] Fedatto-Junior Z, Ishii-Iwamoto EL, Caparroz-Assef SM, Vicentini GE, Bracht A, Kelmer-Bracht AM (2002). Glycogen levels and glycogen catabolism in livers from arthritic rats. Mol Cell Biochem.

[b8-cpath-1-2008-069] Franz MJ (2000). Protein Controversies in Diabetes. Diabetes Spectrum.

[b9-cpath-1-2008-069] Graf J, Haussinger D (1996). Ion transport in hepatocytes: mechanisms and correlations to cell volume, hormone actions and metabolism. J Hepatol.

[b10-cpath-1-2008-069] Holt SHA, Brand Miller JC, Petocz P (1997). An insulin index of foods; the insulin demand generated by 1000-kJ portions of common foods. Am J Clin Nutr.

[b11-cpath-1-2008-069] Haussinger D (1996). The role of cellular hydration in the regulation of cell function. Biochem J.

[b12-cpath-1-2008-069] Jequier E, Tappy L (1999). Regulation of body weight in humans. Physiological Reviews.

[b13-cpath-1-2008-069] Jeor STS, Howard BV, Prewitt E, Bovee V, Bazzarre T, Eckel RH (2001). Dietary Protein and Weight Reduction A Statement for Healthcare Professionals From the Nutrition Committee of the Council on Nutrition, Physical Activity, and Metabolism of the American Heart Association. Am Heart Assoc.

[b14-cpath-1-2008-069] Jiang G, Zhang BB (2003). Glucagon and regulation of glucose metabolism. Am J Physiol.

[b15-cpath-1-2008-069] Jimenez-Gancedo B, Agis-Torres A, Lopez-Oliva ME, Munoz-Martinez E (2004). Dietary protein concentration correlates in a complex way with glucose metabolism and growth performance in pregnant rats. Domestic Animal Endocrinology.

[b16-cpath-1-2008-069] Johnstone AM (1996). Effect of overfeeding macronutrients on day-to-day food intake in man. Eur J Clin Nutr.

[b17-cpath-1-2008-069] Kumar V, Cotran RS, Robbins SL (2000). Basic Pathology.

[b18-cpath-1-2008-069] Kuwajima M, Newgards CB, Foster DW, McGarry ID (1986). The glucose phosphorylating capacity of liver as measured by three independent assays. J Biol Chem.

[b19-cpath-1-2008-069] Lewis GF, Zinman B, Groenewoud Y, Vranic M, Giacca A (1996). Hepatic glucose production is regulated both by direct hepatic and extrahepatic effects of insulin in humans. Diabetes.

[b20-cpath-1-2008-069] Ling C, Kindblom J, Wennbo H, Billig H (2001). Increased resistin expresssion in the adipose tissue of male prolactin transgenic mice and in male mice with elevated androgen levels. FEBS lettters.

[b21-cpath-1-2008-069] Manninen AH (2004). High—protein weight loss diets and purported adverse effects: Where is the evidence?. J. Sport Rev. Nutr..

[b22-cpath-1-2008-069] Myers SR, Diamond MP, Adkins-Marshall BA, Williams PE, Stinsen R, Cherrington AD (1991). Effects of small changes in glucagon on glucose production during a euglycemic, hyperinsulinemic clamp. Metabolism.

[b23-cpath-1-2008-069] Nomura F, Ohnishi K, Ochiai T, Okuda K (1987). Obesity-related nonalcoholic fatty liver: CT features and follow-up studies after low-calorie diet. Radiology.

[b24-cpath-1-2008-069] Palmer M, Schaffner F (1990). Effect of weight reduction on hepatic abnormalities in overweight patients. Gastroenterology.

[b25-cpath-1-2008-069] Radziuk J (1982). Sources of carbon in hepatic glycogen synthesis during absorption of an oral glucose load in humans. Fed Proc.

[b26-cpath-1-2008-069] Rebrin K, Steil GM, Getty L, Bergman RN (1995). Free fatty acid as a link in the regulation of hepatic glucose output by peripheral insulin. Diabetes.

[b27-cpath-1-2008-069] Reeves PG, Nulsen FH, Fahey GC (1993). AIN-93, Purified diets for laboratory rodents: final report of the American Institute of Nutrition adhoc Writing Committee on the reformulation of the AIN-76, a rodent diet. J Nutr.

[b28-cpath-1-2008-069] Schneider K, Laube H, Linn T (1996). A diet enriched in protein accelerates diabetes manifestation in NOD mice. Acta Diabetol.

[b29-cpath-1-2008-069] Shuldiner AR, Yang R, Gong DW (2001). Resistin, Obesity, and Insulin Resistance-The emerging role of the adipocyte as an endocrine organ. N. Engl. J. Med..

[b30-cpath-1-2008-069] Silva SM, Migliorini RH (1990). Effects of starvation and refeeding on energy-linked metabolic process in the turtle. Comp. Biochem. Physiol..

[b31-cpath-1-2008-069] Solomon EP, Berg LR, Martin DW (1999). Biology.

[b32-cpath-1-2008-069] Steppan CM, Bailey ST, Bhat S, Brown EJ, Banerjee RR, Wright CM, Patel HR, Ahima RS, Lazar MA (2001). The hormone resistin links obesity to diabetes. Nature.

[b33-cpath-1-2008-069] Steppan CM, Lazar MA (2002). Resistin and obesity-associated insulin resistance. Trends Endocrinol. Metab..

[b34-cpath-1-2008-069] Ulusoy E, Eren B (2006). Histological changes of liver glycogen storage in mice (Mus musculus) caused by high-protein diets. Histol Histopathol.

[b35-cpath-1-2008-069] Uysal KT, Wiesbrock SM, Marino MW, Hotamisligil GS (1997). Protection from obesity- induced insulin resistancein mice lacking TNF–function. Nature.

[b36-cpath-1-2008-069] Yudkin J (1960). The treatment of obesity by the high fat diet. Lancet.

